# Measurement Properties of the Chinese Version of the Youth Quality of Life Instrument–Weight Module (YQOL-W)

**DOI:** 10.1371/journal.pone.0109221

**Published:** 2014-09-30

**Authors:** Xiao-Ying Jiang, Hong-Mei Wang, Todd C. Edwards, Ying-Ping Chen, Yi-Ran Lv, Donald L. Patrick

**Affiliations:** 1 Institute of Social Medicine and Family Medicine, School of Medicine, Zhejiang University, Hangzhou, Zhejiang, China; 2 Department of Health Services, University of Washington, Seattle, Washington, United States of America; University of Stellenbosch, South Africa

## Abstract

**Background:**

Childhood obesity is a growing public health concern in China. It not only compromises physical health, but also has negative impacts on psychosocial well-being. As obesity rates increase, finding out what the perceptions of Chinese youth are regarding their weight is important for intervention planning and evaluation. However, there is a paucity of available obesity-specific instruments for children and adolescents in China and youth weight-specific quality of life (QOL) has been little reported. This study aimed to evaluate the measurement properties of the Chinese version of the Youth Quality of Life Instrument – Weight Module (YQOL-W).

**Methods:**

The Chinese version of the YQOL-W was administered to 840 youth aged 11–18 from nine schools. Measurement properties including measurement model, reliability, validity and burden were evaluated.

**Results:**

Confirmatory factor analysis showed that a three-factor model had acceptable model fit. The instrument had robust internal consistency reliability with Cronbach's α ranging from 0.84 to 0.96 and acceptable test-retest reliability with the intraclass correlation coefficients (ICCs) all higher than 0.7. The standard error of measurement (SEM) values for the Self, Social and Environment factors and total score were 10.352, 9.526, 12.086 and 8.425, respectively. The small real differences (SRDs) for the Self, Social and Environment factors and total score were 28.675, 26.387, 33.478, and 23.337, respectively. The Pearson's correlation coefficients between the YQOL-W and the PedsQL4.0 General Core Scales were stronger between comparable dimensions than those between less comparable dimensions, demonstrating convergent and discriminant evidence of construct validity. Significant differences were found in subscale and total scores across weight status, age and genders (*P*<0.01), supporting the known-groups validity of the instrument.

**Conclusion:**

The Chinese version of the YQOL-W has acceptable measurement properties and can be used to assess the weight-specific QOL of children and adolescents in China.

## Background

Probably owing to socioeconomic transition and increasing adoption of a lifestyle consisting of high-fat prepared foods and sedentariness, China has entered an epidemic stage of childhood obesity [1], [2], [3], [4]. According to data from the National Survey on Students' Physical Fitness and Health in 2010, the prevalence of obesity was 13.33, 5.64, 7.83, 3.78 percent for urban boys, urban girls, rural boys and rural girls respectively. This is 1.94, 0.63, 2.76, 1.15 percent higher than the prevalence in 2005 respectively and comparable with developed countries in some subgroups such as urban and high-income groups [5].

Childhood overweight and obesity has serious health consequences [6], [7], [8]. Overweight and obese children are likely to remain obese as adults and are at increased risk for obesity comorbidities like type 2 diabetes and cardiovascular diseases at a younger age, leading to premature mortality and long-term morbidity [4], [9], [10]. The most common short-term consequences of childhood obesity are primarily psychosocial however, including teasing, exclusion, and discrimination [6], [11], [12]. A recent review found that children and adolescents with obesity have reduced quality of life (QOL) compared with their lean counterparts [13]. One study reported that obese children and adolescents have a QOL similar to children and adolescents diagnosed with cancer [12].

The development of QOL instruments for children and adolescents, particularly disease-specific questionnaires, has continued apace in recent years. Solans et al. listed 27 conditions covered by disease-specific instruments developed for children and adolescents between 1980 and 2006, with asthma, cancer and epilepsy identified as most frequent conditions [14]. The majority of existing instruments focus primarily on functional status or performance of daily activities however, and there is a shortage of instruments that tap perceptions or feelings and involve children directly in critical stages of instrument development [14], [15], [16].

Youth quality of life research in China is emerging at present. Limited studies have used established youth quality of life instruments to examine the impacts of pediatric diseases on children and adolescents. Most of them used translations of English instruments without qualitative research and validation and some have used QOL instruments designed for adults [17]–[22]. Weight-specific quality of life among youth has been little reported to date. In direct response to the need for a measure of health-related quality of life for children and adolescents with obesity in China, a careful translation and psychometrically robust measurement is required. The Youth Quality of Life Instrument-Weight Module (YQOL-W) which has been comprehensively developed by the Seattle Quality of Life Group (SeaQoL) at the University of Washington, appears to meet all of psychometric standards compared with other weight-specific patient reported outcome (PRO) instruments [16], [23]. The YQOL-W is unique in that it was developed through a series of in-depth interviews with African American, Mexican American, and white youth rather than expert opinion and takes into account culturally-sensitive issues surrounding weight and quality of life. The YQOL-W module consists of 21 weight-specific items corresponding to three domains of conceptual framework for QOL in youth by Edwards et al., briefly Self, Social and Environment [24], [25].

The Chinese version of the YQOL-W has been primarily developed through linguistic validation and qualitative research phases [26], therefore, the purpose of this study was to evaluate the measurement properties of the Chinese version of the YQOL-W and examine whether it can be used to assess the weight-specific QOL of children and adolescents in China.

## Materials and Methods

### Ethics statement

This study was approved by Zhejiang University School of Medicine Ethics Committee. The study's purpose and all the procedures involved were explained in a youth-friendly and understandable way to all potential participants. Those who had verbal consent were followed up. Only youth providing with informed written consent, both individual and parental/guardian were included in the study. All data were analyzed anonymously.

### Sample and study design

The study was conducted in Hangzhou, Zhejiang Province of China in 2012. In order to ensure breadth and representativeness of the obtained sample, the recruitment cells were settled in advance. The sample was stratified such that approximately equal numbers of participants were recruited with respect to gender (male, female), age (11–18 YD), recruitment community (urban, migrant, rural) and body mass index (normal, overweight, obese), leading to a total of 36 recruitment cells. The participants were recruited from 9 schools and potential participants were randomly chosen by the study coordinator based on latest anthropometric examination. They were informed of the study with a flyer from their teachers. For those who had verbal consent, teachers handed written informed consents (individual and parents/guardians), as well as self screening sheets to them, and recruitment didn’t stop until the recruitment quotas were saturated. Eventually, a total of 840 potential participants who had returned written informed consents and passed screening were recruited in the field study.

Participants in this study were limited to 11–18 years of age. Youth had to able to read and comprehend Chinese at least at a 5th-grade reading level and to write sufficiently well to respond to the battery of measures. Potential participants were approached except for conditions, which were confirmed from self screening: 1) pregnant or nursing; 2) currently receiving any psychotropic medication; 3) disease history of anorexia nervosa, bulimia, major depression, panic disorders, psychosis, bi-polar disorders; 4) with a life-threatening illness; 5) co-morbid physical disabilities, long-term health problems, or mental health disorders that had a greater impact on their life than their weight. During the procedure, it was emphasized that participation in the study was voluntary and had no effects on school marks. All information given to the researchers was kept strictly confidential. Participants received a gift for attendance.

The participants were asked to fill the questionnaires including personal demographic statistics, the Chinese versions of the YQOL-W module and PedsQL4.0 Generic Core Scales. All questionnaires were administered with minimal supervision by the study coordinator. After the questionnaire was administered, each participant's height and weight were measured following a standard protocol. Body mass index(BMI; kg/m^2^) was calculated and the weight status of participants were sorted according to BMI cut-off points for screening overweight and obesity in Chinese children and adolescents which was established by Group of China Obesity Task Force [27]. A subset of 90 participants was selected at random to complete the YQOL-W module 7-10 days after the baseline survey to assess test-retest reliability.

### Instruments

#### The Chinese Version of the YQOL-W

According to methodology of the YQOL-W Translation Manual, the linguistic validation of the instrument was strictly conducted using forward translation, backward translation, and participant testing. We further checked conceptual equivalence and guided construction of culture specific items using inductive qualitative methods. Twenty two in-depth semi-structured interviews with youth ages 11–18 were conducted to explore their areas of life, goals, concerns, and worries associated with weight and weight management. Using thematic analysis to identify, categorize and classify themes in the data, five culture specific new items were elicited and added to the instrument. The Chinese Version of the YQOL-W was used for measurement properties evaluation in this study [26].

#### The Chinese Version of the PedsQL4.0 General Core Scales

The Pediatric Quality of Life Inventory Measurement Models (PedsQL) was first developed by Varni et al in 1999. It includes a general core scale and several disease specific modules. The PedsQL General Core Scales has 23 items grouped into four subscales: Physical Functioning (8 items), Emotional Functioning (5 items), Social Functioning (5 items) and School Functioning (5 items).It was reported that the Internal consistency reliability for the total scale and subscales were all over 0.70 and could distinguish between healthy children and pediatric patients with acute or chronic health conditions, demonstrating robust reliability and validity of the PedsQL 4.0 Generic Core Scales [28]. It was the most commonly used patient-reported outcome instrument for children and adolescents with obesity [23]. The Chinese version of the PedsQL4.0 Generic Core Scales has acceptable measurement properties [29], therefore we include it in our study for examining the convergent and discriminant validity of the Chinese YQOL-W.

### Statistical analysis

All data were double entered, missing values were checked against the paper and pencil surveys and inconsistencies were corrected before analysis. After data cleaning, the endorsements of participants were linearly transformed into scores according to respective scoring methods with higher scores indicating better QOL.

Descriptive analysis was used for reporting the demographic statistics of the participants. Continuous variables were displayed as mean, standard deviations, skewness and kurtosis. Categorical variables were presented as observed frequencies and proportions.

Feasibility was determined by average completion time of the Chinese Version of the YQOL-W and the percentage of missing value for each item. Floor or ceiling effects are considered to be present if more than 15% of respondents achieved the lowest or highest possible score, respectively [30].

Exploratory factor analysis (EFA) was applied to examine the latent constructs of the instrument. Bartlett's test of sphericity coefficient and Kaiser-Meyer-Olkin (KMO) test of sampling adequacy were computed to determine whether there were a sufficient number of significant correlations among the items to justify EFA. Principal components analysis was used to extract principal factors with several criteria including Guttman's weakest lower bound, the percent of variance explained, scree plots and the interpretability of the factors [31].

Orthogonal and oblique factor rotations were computed after establishing the number of factors to extract. Following each rotation, the factor structure was examined to find the rotation method that produced the most interpretable simple structure. Items without a loading of 0.40 or higher on any factor and items with loadings of 0.40 or higher on multiple factors were considered for elimination from item pool [16].

Confirmatory factor analysis (CFA) was conducted to investigate the factor structure. The aim of CFA was to test the hypothesis that there existed a relationship between the observed variables (items) and their underlying latent constructs (subscales). χ^2^ test was used to evaluate the model adequacy. χ^2^/df was also used, for χ^2^ test was sensitive to sample size [32]. Common goodness-of-fit indices, namely, Goodness of Fit index (GFI), Root Mean Square Error of Approximation (RMSEA), and Standardized Root Mean Square Residual (SRMR) were used to assess the fitness [33], [34].

Reliability and validity of the Chinese version of the YQOL-W were assessed according to established guidelines [35]. Internal consistency reliability was determined using Cronbach's Alpha Coefficient, values greater than 0.7 are considered to be appropriate for comparing different groups [36]. Test-retest reliability was determined using the intraclass correlation coefficient (ICC). An ICC greater than 0.70 is considered appropriate for group comparison [37]. The standard error of measurement (SEM_agreement_) was used to assess variability, i.e., the absolute measurement error [30], [38]. The smallest real difference (SRD) was calculated (1.96×
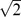
×SEM) to estimate the smallest detectable change in individual patients that can be distinguished from measurement error [39].

Construct validity was evaluated by convergent, discriminant and known groups evidence. First, Pearson's correlation was used to measure the association between the Chinese Version of the YQOL-W and the Chinese Version of the PedsQL4.0. It was expected that comparable dimensions, e.g., YQOL-W social domain and PedsQL social functioning, would correlate better, compared with less comparable dimensions, such as YQOL-W social domain and PedsQL school functioning. Pearson's correlation coefficients below 0.30 indicated a weak correlation, 0.30–0.49 moderate and 0.50 or above strong [40]. Second, we hypothesized that YQOL-W scores will decrease as BMI values increase [12], [41]. We also expected that the QOL will be lower for girls and those with low incomes and increasing age [13],[42],[43],[44]. Group differences were assessed using ANOVA or *t-*test.

All statistical analyses were carried out using SPSS version 17 and Amos 16.0 software (SPSS Inc., Chicago, IL, USA), a probability of less than or equal to0.05 was considered as statistically significant.

## Results

The average completion time of the Chinese Version of the YQOL-W was 5-8 minutes. Among the 840 participants enrolled, complete data for the YQOL-W were available for 814 participants (96.9%) and analyzed for the present study. Of the 814 respondents included in this study, 51.8% were between 11 and 14 years of age and 48.2% were between 15 and 18 years of age, 50.6% were girls. About thirty-three percent of the respondents had a normal BMI, 41.8% were overweight and 25.7% were obese (Table 1).

**Table 1:** Demographic Characteristics of the Sample(*n* = 814).

	N^a^	%
**Age in years**		
11–14	422	51.8
15–18	392	48.2
**Gender**		
Male	402	49.4
Female	412	50.6
**Ethnicity**		
Han nationality	790	97.1
Non-Han	10	1.1
**Number of family members**		
2	8	1.0
3	348	42.8
4	245	30.1
≥5	196	24.1
**Father's education**		
Middle school or less	386	47.4
High school or vocational training	268	32.9
Some college	63	7.7
College	70	8.6
Masters or higher	11	1.4
**Mother's education**		
Middle school or less	471	57.9
High school or vocational training	225	27.6
Some college	66	8.1
College	37	4.5
Masters or higher	11	1.4
**Annual household income**		
<60,000 Yuan	316	38.8
>60,000 Yuan	448	55.0
**Weight status**		
Normal	265	32.6
Overweight	340	41.8
Obese	209	25.7
**Recruitment community**		
Urban community	264	32.4
Rural community	275	33.8
Migrant community	275	33.8

a. Sample sizes within characteristics may not sum to *n* = 814 due to missing values

### Item descriptive statistics

Item means ranged from 7.27 to 9.24 (Table 2). The percent of responses at the floor (score = 0) ranged from 0.9 to 9.2%, and the percent of responses at the ceiling (score = 10) ranged from 35.3 to 76.4%, indicating that item responses tended to bunch at the upper end of the QOL continuum, ceiling effect was notable. Item skewness ranged from −3.14 to −0.93, and item kurtosis ranged from −0.11 to 10.27, demonstrating negativity skewed distribution.

**Table 2:** Descriptive statistics for the Chinese version of the YQOL-W item pool (*n* = 814).

Item no. and brief item content	Mean	SD	%Floor	%Ceiling
1 Feel depressed	7.27	2.94	4.8	35.3
2 Feel ashamed	7.95	2.74	3.7	47.3
3Uncomfortable with skinny people	8.14	2.75	4.2	52.1
4 Hide my body	7.65	3.16	6.3	49.3
5 Feel unattractive	8.25	2.60	2.7	54.9
6 Avoid photographs	8.66	2.49	2.2	67.9
7 Embarrassed to exercise	8.65	2.29	1.6	62.4
8 Embarrassed to eat	9.03	2.00	1.2	71.5
9 Avoid being noticed	8.75	2.32	1.8	66.3
10 Worry what people saying	7.97	2.79	3.9	48.5
11 Uncomfortable at social events	8.61	2.35	1.5	61.9
12 Feel like a loser	8.43	2.62	3.1	58.8
13 Uncomfortable moving	8.74	2.18	0.9	62.4
14 Avoid swim suits	7.46	3.34	9.2	47.1
15Hard finding girlfriend or boyfriend	8.66	2.45	2.1	68.4
16 People stare	8.8	2.29	2.0	66.0
17 Not included	9.24	1.86	1.4	76.4
18 Hard getting job	8.55	2.54	3.3	62.7
19 Difficult wearing cloths	7.60	3.20	6.9	48.0
20 Difficult finding clothes	7.84	3.00	4.7	50.9
21 Hard to exercise	8.32	2.60	2.6	57.6
22 Feel not as good as others	8.78	2.25	1.8	64.3
23 Feel upset	7.68	2.98	4.9	44.0
24 Difficult getting a good grade in physical education class	7.47	3.30	8.1	48.8
25 Uncomfortable around strangers	8.45	2.51	1.8	60.4
26 Worry others limiting my opportunities	8.52	2.44	1.8	59.3

### Measurement model adequacy

Exploratory factor analysis was conducted to examine the latent constructs of the instrument. The result of Barlett's test of sphericity (χ^2^ = 15710.664, df = 325, *P*<0.001) and the KMO statistic (0.964), supported for conducting exploratory factor analysis.

The first four factors was extracted from principal component analysis, meeting the Kaiser retention of eigenvalues greater than 1, that were 13.67,1.58,1.11 and 1.03 respectively, accounting for 66.89% of the accumulative variance. The scree plot also showed that the first inflection point was at the fourth eigenvalues, suggesting that four factors could be extracted [31].

Factor rotation using orthogonal rotation (Varimax) and oblique rotation (Promax) were administrated to extract four factors. After orthogonal and oblique rotations, the results indicated that the oblique rotations took advantage of grouping items with similar content together due to correlated domain scores. The four-factor models with oblique solution tended to group items into three main factors and a small factor with two items (item 21 and 24) relevant to physical activity. Because scale developers recommend against factors with few than three items [16], besides, item 23 (I feel upset when people my age tease me about my weight) didn’t have a loading of 0.4 or higher on any factor in the four-factor models, we considered that four-factor models might not be the most readily interpretable and theoretically sensible pattern of the results. After the elimination of item 23, three-factor solutions was inspected. Item 7 and 13 were found that they didn’t have a loading higher than 0.4 on any factor. Item content and their importance to the measurement of the relevant construct were reviewed. Item 7 (Because of my weight I am embarrassed to exercise around other people) was found unimportant to Chinese youth for they got used to exercise with their classmates and item 13 (Because of my weight my body feels uncomfortable when I move around) was found perceptual ambiguity in Chinese context (Quite a lot comprehended it as emotional unease), therefore a decision was made to delete both of them.

After dropping the items 23, 7 and 13, the factor analysis with oblique solution automatically achieved three-factor model, all the items yielded to their latent factors with loadings above 0.4, respectively. The final factor loadings for the three-factor model are reported in Table 3. The Self factor contained 7 items with factor loading ranging from 0.42–0.96, the Social factor contained 12 items with factor loading ranging from 0.46–1.00 and the Environment factor had 4 items with factor loading ranging from 0.59–0.92. Item 5, item 6 belonging to Social domain and item 14 belonging to Environment domain in the original scale were grouped into Self domain after the factor analysis.

**Table 3:** Exploratory factor analysis and confirmatory factor analysis for the YQOL-W three-factor model.

Item no. brief item content		YQOL-W		
		Self	Social	Environment
1 Feel depressed		0.96		
2 Feel ashamed		0.90		
3 Uncomfortable with skinny people		0.84		
4 Hide my body		0.90		
5 Feel unattractive		0.58		
6 Avoid photographs		0.52		
14 Avoid swim suits		0.42		
8 Embarrassed to eat			0.74	
9 Avoid being noticed			0.57	
10 Worry what people saying			0.52	
11 Uncomfortable at social events			0.66	
12 Feel like a loser			0.49	
15 Hard finding girlfriend or boyfriend			0.46	
16 People stare			0.74	
17 Not included			1.00	
18 Hard getting job			0.62	
22 Feel not as good as others			0.72	
25 Uncomfortable around strangers			0.60	
26 Worry others limiting my opportunities			0.68	
19 Difficult wearing cloths				0.59
20 Difficult finding clothes				0.59
21 Hard to exercise				0.84
24 Difficult getting a good grade in physical education class				0.92
**Goodness-of-Fit Indices**				
CMIN (χ^2^)	χ^2^/df	GFI	RMSEA	Standard RMR
900.598	4.003	0.904	0.061	0.073

We further evaluated the three-factor model using confirmatory factor analysis. Generalized least squares (GLS) estimation was conducted due to skewness of data distribution [45]. The Goodness-of-Fit statistics of the three-factor model based on the original scaling structure are also presented in Table 3. χ^2^/df was 4.00, a little more than 3.00. GFI values were greater than 0.90, RMSEA within a maximum value of 0.08, but Standard RMR was a little higher than 0.05.

### Internal consistency reliability

Table 4 presented item-scale correlations and Cronbach's alpha coefficients of the YQOL-W. The average, smallest and largest inter-item correlations were 0.50, 0.26 and 0.84, respectively. Item-scale correlations for the Self, Social and Environment factors were 0.58–0.81, 0.62–0.81 and 0.59–0.74, respectively. Cronbach's alpha coefficients for the Self, Social, and Environment factors was 0.90, 0.94, and 0.84 respectively, and dropping either item in the belonged factor had a small negative effect on respective coefficient alpha. For the total score, Cronbach's alpha coefficient was 0.96, and dropping either item had a small negative effect on coefficient alpha with a few exceptions (data not shown).

**Table 4:** Internal consistency reliability analysis of the YQOL-W (*n* = 814).

Social and item content (Item number)	Item-scale correlations	Cronbach's alpha coefficients if item deleted
**Self**		0.90
1 Feel depressed	0.76	0.88
2 Feel ashamed	0.81	0.87
3 Uncomfortable with skinny people	0.72	0.88
4 Hide my body	0.77	0.88
5 Feel unattractive	0.67	0.89
6 Avoid photographs	0.66	0.90
14 Avoid swim suits	0.58	0.90
**Social**		0.94
8 Embarrassed to eat	0.66	0.93
9 Avoid being noticed	0.68	0.93
10 Worry what people saying	0.78	0.93
11 Uncomfortable at social events	0.81	0.93
12 Feel like a loser	0.70	0.93
15 Hard finding girlfriend or boyfriend	0.62	0.93
16 People stare	0.76	0.93
17 Not included	0.62	0.93
18 Hard getting job	0.69	0.93
22 Feel not as good as others	0.77	0.93
25 Uncomfortable around strangers	0.75	0.93
26 Worry others limiting my opportunities	0.76	0.93
**Environment**		0.84
19 Difficult wearing cloths	0.71	0.78
20 Difficult finding clothes	0.74	0.77
21 Hard to exercise	0.66	0.80
24 Difficult getting a good grade in physical education class	0.59	0.83

### Test–retest reliability

Of the 90 participants included in the retest sample, all returned the retest questionnaire. The interval of test-retest measurement was 7–10 days. The ICCs for the Self, Social and Environment factors and total score were 0.709, 0.721, 0.778 and 0.778, respectively. The SEM values for the Self, Social and Environment factors and total score (all 0–100 scale) were 10.352, 9.526, 12.086 and 8.425, respectively. The SRDs for the Self, Social and Environment factors and total score were 28.675, 26.387, 33.478, and 23.337, respectively.

### Construct Validity

The Pearson's correlation coefficients between the YQOL-W and the PedsQL were stronger between comparable dimensions(e.g., 0.527 between YQOL-W environment domain and PedsQL physical functioning, 0.436 between YQOL-W social domain and PedsQL social functioning) than those between less comparable dimensions (e.g., 0.213, 0.245, 0.245 between YQOL-W self, social and environment domain and PedsQL school functioning, respectively) with a few exceptions, demonstrating convergent and discriminant evidence of construct validity (Table 5).

**Table 5:** Correlations between the YQOL-W and the PedsQL (n = 792).

PedsQL dimensions	YQOL-W			
	Self	Social	Environment	Total score
Physical functioning	0.314	0.394	0.527	0.433
Emotional functioning	0.287	0.325	0.324	0.342
Social functioning	0.304	0.436	0.409	0.423
School functioning	0.213	0.245	0.245	0.257
Total score	0.355	0.441	0.488	0.465

Sample size was 792 due to missing values in the PedsQL Generic Core Scales.

Pearson's correlation, all *P*<0.001.

Table 6 presents the construct validity of the YQOL-W assessed by the known-group method. The One-Way ANOVA suggested significant weight status differences on subscales. And the following pairwise comparison indicated that as BMI values increased, the scores decreased (all *P*<0.01, data not shown). Girls reported significantly lower scores than boys, and older adolescents reported significantly lower scores than younger ones with a few exceptions. However, no significant differences in annual household income were found on any subscale.

**Table 6:** Validity of the YQOL-W assessed by the known-group method (n = 814).

Variables	N	Self factor		Social factor		Environment factor		Total score	
		mean(SD)	t(P)	mean(SD)	t(P)	mean(SD)	t(P)	mean(SD)	t(P)
**Gender**									
Male	402	85.81(19.33)	8.725	89.87(15.70)	5.339	82.54(23.04)	5.116	87.36(16.33)	7.030
Female	412	72.76(23.73)	(<0.001)	83.17(19.18)	(<0.001)	73.74(25.91)	(<0.001)	78.30(20.18)	(<0.001)
**Age in years**									
11–14	422	82.19(9.17)	4.076	87.41(18.30)	1.512	80.80(23.60)	3.247	84.67(18.53)	2.982
15–18	392	75.78(10.91)	(<0.001)	85.48(18.06)	(0.131)	75.16(25.96)	(0.001)	80.73(19.14)	(0.003)
**Annual household income**									
<60,000 Yuan	316	78.59(22.45)	−0.416	85.91(18.14)	−0.664	77.21(25.09)	−0.732	82.17(18.78)	−0.654
≥60,000Yuan	448	79.28(22.90)	(0.667)	86.80(18.47)	(0.507)	78.55(25.02)	(0.464)	83.08(19.15)	(0.514)
**Weight status**									
Normal	265	87.77(18.06)	37.072^ a^	93.80(11.27)	48.221^ a^	90.92(15.12)	89.744^ a^	91.46(12.64)	62.598^ a^
Overweight	340	77.38(22.76)		85.85(18.23)		77.35(24.52)		81.74(19.03)	
Obese	209	70.91(24.06)	(<0.001)	78.21(21.32)	(<0.001)	63.00(26.75)	(<0.001)	73.34(20.47)	(<0.001)

Independent sample t test for gender, age and income group, One-Way ANOVA for weight status.

a. F value for weight status.

## Discussion

The development of the Chinese version of the YQOL-W was to measure the weight-specific quality of life for children and adolescents of 11–18 years age in China. This study assessed the measurement model, reliability, validity and measurement burden of the YQOL-W.

The ceiling effect was notable on the Chinese version of the YQOL-W, which was consistent with the findings of other studies [16], [46]. It could limit the capacity of this tool to accurately detect the differences of quality of life between the adolescents with the best scores and any improvements over time in these areas would not easily be detected. As a consequence, the reliability and responsiveness of the instrument might be reduced [29]. Exploratory factor analysis of 23 items yielded three factors reflecting Social-, Environmental- and Self-related aspects of weight-specific QOL, which fitted into the theoretical expectation [16]. Item 5 (Because of my weight other people think I am unattractive), item 6 (Because of my weight I try to hide behind other people when I get my picture taken) and item 14 (Because of my weight I avoid being seen in a swim suit) redrew to self domain. Chinese youth tended to comprehend these items as self-emotional related concept rather than getting along with others or their opportunities from the environment.

In the current study, CFA was utilized to determine the construct validity of the instrument. The premeditated three factor model demonstrated a little high but still acceptable model fit by χ^2^/df ratios. Our sample size might account for the problem. As Bentler put it, if the data was not normal distribution, a proportion more than 10∶1 was required for sample with free parameters [47]. Given that parameters of 225, our sample size didn’t meet the theoretic expectation. GFI and RMSEA reached adequate values indicating acceptable model fit except Standard RMR.

All the Cronbach's alpha coefficients exceeded the recommended standard of 0.7 for group comparisons, indicating acceptable internal consistency reliability of the Chinese Version of the YQOL-W. Test-retest reliability was determined by computing ICC. All the ICCs were greater than 0.70 which was appropriate for group comparisons. The ICCs not only reflect the degree of agreement between repeated measures, but also the degree to which a measurement instrument can differentiate among individuals [48].In a homogeneous population, more within-subject variance than between-subject variance leads to low reliability [49], [50]. The SEM is fairly sample-independent and more stable [49], [51]. It is useful in the interpretation of change scores when converted to the SRD. Only changes of at least the SRD can be statistically significantly distinguished from measurement error in individual patients [39]. The SEM of the Chinese Version of the YQOL-W (8–12 percent of measurement score) and the SRD (23–33 percent of measurement score) were relatively large in our study, therefore, future study is needed to determine its usefulness for individual monitoring in daily clinical practice [39].Construct validity of the YQOL-W was assessed by convergent, discriminant, and known-group methods. Convergent and discriminant validity is defined as the extent to which one measure correlates with another measure of the same concept [52].Our study demonstrated that comparable dimensions correlate better than those less comparable dimensions. The YQOL-W environment domain was found highly correlated with PedsQL physical functioning, due to half of items in the YQOL-W environment domain deal with physical aspect. While the generic PedsQL 4.0 was probably the most commonly used PRO instrument in children with obesity, many health domains were found in the obesity-specific instruments that are not measured by the PedsQL 4.0 [23]. More evidence of convergent and discriminant validity could be expected between the YQOL-W and another disease-specific instrument with comparable conceptual approach to measurement, QOL other than Functioning, disability and health (FDH) approach.

Known-group method is determined by the degree to which one measure can differentiate between groups thought to differ in terms of marker variables [52], [53]. In this study, hypotheses of weight status, gender and age differences on scales were verified as expected. However, our study reported that no significant differences in annual household income were found on any subscale. Janicke et al. [43] found there was a significant relationship between family income and parent-reported QOL physical summary score in overweight youth. Since the YQOL-W was reported by the youth, this might link to the disparities.

Certain limitations should be considered within this study. First, our sample was selected from the children and adolescents of 11–18 years old in Hangzhou, China, were not representative of target population in other areas. Therefore, cautions are recommended if the results were extended to other regions in this country. Second, a larger sample size will be helpful to achieve the stability for the confirmatory factor analysis. Finally, it was rarely possible to evaluate all the measurement properties of the instrument in a single study. Ability to detect weight change over time and interpretability of YQOL-W scores need to be further examined in longitudinal studies.

## Conclusions

The Chinese version of the YQOL-W presents acceptable measurement properties in urban, rural and immigrant community sample of obese, overweight, and normal weight youth of 11–18 years age in Hangzhou, China. The instrument can be used to assess the weight-specific QOL of children and adolescents in China. Future research will address the ability of the YQOL-W to detect weight change over time and interpretability of YQOL-W scores.
